# Information processing correlates of a size-contrast illusion

**DOI:** 10.3389/fpsyg.2014.00142

**Published:** 2014-02-19

**Authors:** Jason M. Gold

**Affiliations:** Department of Psychological and Brain Sciences, Indiana University, BloomingtonIN, USA

**Keywords:** visual illusion, response classification, noise, efficiency, ideal observer

## Abstract

Perception is often influenced by context. A well-known class of perceptual context effects is perceptual contrast illusions, in which proximate stimulus regions interact to alter the perception of various stimulus attributes, such as perceived brightness, color and size. Although the phenomenal reality of contrast effects is well documented, in many cases the connection between these illusions and how information is processed by perceptual systems is not well understood. Here, we use noise as a tool to explore the information processing correlates of one such contrast effect: the Ebbinghaus–Titchener size-contrast illusion. In this illusion, the perceived size of a central dot is significantly altered by the sizes of a set of surrounding dots, such that the presence of larger surrounding dots tends to reduce the perceived size of the central dot (and *vise versa*). In our experiments, we first replicated previous results that have demonstrated the subjective reality of the Ebbinghaus–Titchener illusion. We then used visual noise in a detection task to probe the manner in which observers processed information when experiencing the illusion. By correlating the noise with observers' classification decisions, we found that the sizes of the surrounding contextual elements had a direct influence on the relative weight observers assigned to regions within and surrounding the central element. Specifically, observers assigned relatively more weight to the surrounding region and less weight to the central region in the presence of smaller surrounding contextual elements. These results offer new insights into the connection between the subjective experience of size-contrast illusions and their associated information processing correlates.

## INTRODUCTION

Context can often exert a significant influence on perception. Famous examples of context effects include crowding (Bouma, 1970), word superiority effects (Johnston and Mcclelland, 1974), configural superiority effects (Pomerantz et al., 1977), the kinetic depth effect (Wallach and O’Connell, 1953), point-light biological motion perception (Johansson, 1973), Gestalt grouping and perceptual organization (Koffka, 1935), and visual completion (Kanizsa, 1979). Another related category of context effects involves the perceptual consequences of introducing contrast between elements within a display. Examples of contrast effects include lightness and brightness contrast illusions (Cornsweet, 1970; Adelson, 1993; Gilchrist et al., 1999), color contrast illusions (Jameson and Hurvich, 1964; Lotto and Purves, 2000), and size-contrast illusions (Coren and Girgus, 1978).

In the cases of lightness, brightness, and color contrast illusions, the underlying physiological and information processing mechanisms that mediate these effects have been studied extensively (e.g., Jameson and Hurvich, 1964; Cornsweet, 1970; Adelson, 1993; Lotto and Purves, 2000). In the case of size-contrast illusions, most research has focused on exploring the conditions that are most favorable for inducing the illusions (e.g., Girgus et al., 1972; Coren and Girgus, 1978; Jaeger, 1978; Weintraub, 1979; Weintraub and Schneck, 1986; Rose and Bressan, 2002; Roberts et al., 2005; Daneyko et al., 2011), demonstrating the behavioral impact of the illusions in various tasks (e.g., Jaeger, 1978; Pavlova and Sokolov, 2000; Haffenden et al., 2001; Rose and Bressan, 2002; Westwood and Goodale, 2003; Handlovsky et al., 2004; Muller and Busch, 2006; Im and Chong, 2009; Sperandio et al., 2010, 2012), or using the illusions as research tools to understand various aspects of perceptual processing, such as whether apparent size is coded in pre-attentive vision (Busch and Muller, 2004) and whether there are two separate visual processing streams (e.g., Aglioti et al., 1995; Milner and Goodale, 1995; Goodale and Humphrey, 1998).

**FIGURE 1:**
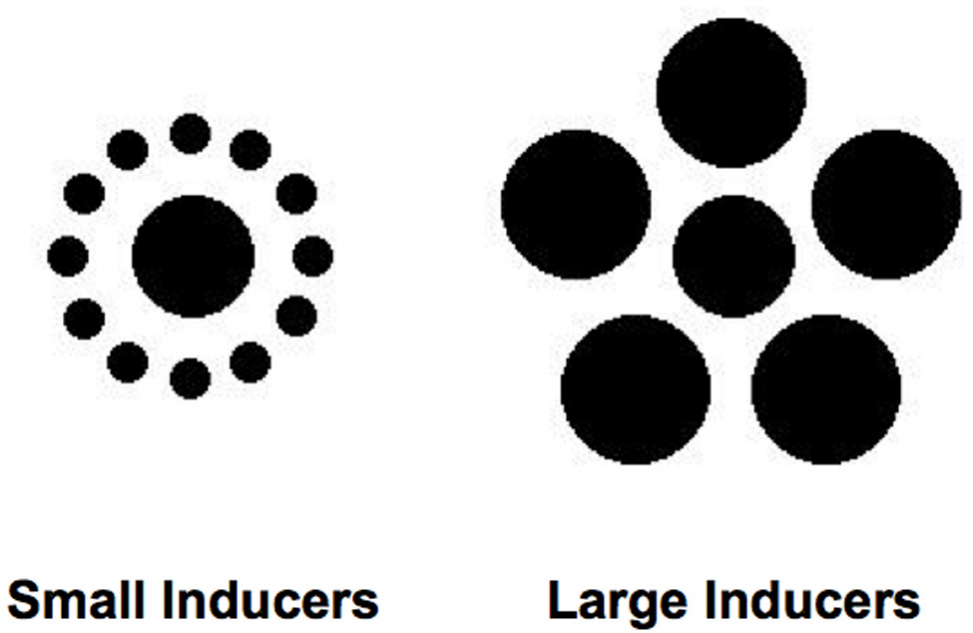
**Ebbinghaus–Titchener figures used as stimuli in the experiments.** The central dots of the figures are the same physical diameter.

One size-contrast illusion, the Ebbinghaus–Titchener illusion (Titchener, 1901), has been used most extensively in this research. **Figure 1** shows the canonical form of the Ebbinghaus–Titchener illusion. When most observers view these figures, the central dot is judged to be significantly larger when encircled by smaller dots (left side of **Figure 1**) than when surrounded by larger dots (right side of **Figure 1** ). The magnitude of this effect has been shown to depend upon many additional factors, including the distance between the central dot and the surrounding dots, the number and density of surrounding dots, the similarity between the central and surrounding dots, and even the age, sex, and culture of the observer (Massaro and Anderson, 1971; Coren and Girgus, 1978; Weintraub, 1979; Weintraub and Schneck, 1986; Choplin and Medin, 1999; Phillips et al., 2004; Roberts et al., 2005; de Fockert et al., 2007; Daneyko et al., 2011). Nevertheless, the subjective experience of the Ebbinghaus–Titchener illusion is quite reliable and robust for most observers under a wide range of conditions.

Despite the extensive amount of research that has involved this size-contrast effect, the connection between the subjective experience of the illusion and the specific manner in which information is processed by the visual system is not well understood. There are may possible ways in which the experience of the illusion might map on to how observers make use of information when performing tasks that rely upon the part of the stimulus that is perceptually altered by the presence of the inducing elements. For example, observers might make use of a relatively larger region of the central portion of the stimulus in the presence of smaller inducing elements. Another possibility is that observers might differentially rely upon the regions within and immediately surrounding the central dot, depending upon the size of the inducing elements. Alternatively, there may be no little or connection between observers’ subjective experience of the illusion and how they make use of information in tasks involving these stimuli.

Thus, the goal of the current study was to directly address this question by exploring the underlying information processing correlates associated with the perception of the Ebbinghaus–Titchener size-contrast illusion in a perceptual task. We approached this problem by first measuring and verifying the traditional subjective size-contrast effects associated with the illusion. We then employed these same stimuli to be used within the context of a performance-based rather than a subjective judgment task. Specifically, we had observers perform a simple detection task with the central dot of Ebbinghaus–Titchener figures under conditions of varying context (i.e., in the presence of larger or smaller surrounding dots). We chose a detection task as a starting point because of its relative simplicity. Observers performed this task with stimuli that were embedded in high contrast pixel noise, which allowed us to measure the impact of context on two related aspects of information processing: (a) the overall *efficiency* with which observers make use of information (i.e., their performance relative to a statistically optimal or *ideal observer*); and (b) the perceptual strategy or “template” used by observers, determined by correlating the noise shown across trials with observers’ decisions (i.e., *response classification*). A similar approach has been used successfully to explore the information processing correlates associated with brightness–contrast context effects (Shimozaki et al., 2005).

## MATERIALS AND METHODS

### PARTICIPANTS

Three observers (two males, mean age 20) participated in both experiments. All were paid for their participation, gave written consent and had normal or corrected-to-normal visual acuity (self-reported). Two were naïve to the purposes of the experiments and one was a paid laboratory research assistant (observer PM). The study was approved by the Indiana University Human Research Protection Program.

### APPARATUS

All stimuli were displayed on a Sony Trinitron G520 CRT monitor (resolution: 1024 pixels × 768 pixels; size: 38.25 cm × 28.5 cm; refresh rate: 85 Hz). The display was calibrated using a Minolta LS-100 photometer. The background was fixed at a luminance of 85 cd/m^2^, and the CRT provided the only source of illumination during the experiment. Viewing distance was fixed at 130 cm using a head/chin rest. All aspects of the experiment, including stimulus generation, presentation, and data analysis, were carried out within the MATLAB programming environment (version 7.1) using in-house software and the extensions provided by the psychophysics toolbox (Brainard, 1997).

### STIMULI

Stimuli consisted of a central dot (45 pixels in diameter, 0.74°) surrounded by a series of “inducing” dots (**Figure 1**). In the Small Inducers condition, there were 12 surrounding dots of equal size (15 pixels in diameter, 0.25°), equidistant from the central dot (45 pixels from the midpoint of each inducer to the midpoint of the central dot, 0.74°) and equally spaced around the perimeter of a virtual circle centered upon the central dot. In the Large Inducers condition, there were five dots of equal size (55 pixels in diameter, 0.9°), equidistant from the central dot (60 pixels from the midpoint of each inducer to the midpoint of the central dot, 0.98°) and equally spaced around the perimeter of a virtual circle centered upon the central dot. In Experiment 1, a central dot of variable size with no surrounding inducing elements was also used to obtain estimates of perceived size.

All stimuli were defined in terms of contrast, with the contrast at each pixel defined as the luminance value relative to the background luminance (i.e., *L*_pixel_ - *L*_background_)/*L*_background_). Stimuli were negative in contrast (i.e., darker than the background). In Experiment 1, each pixel of the entire stimulus was set to the maximum displayable negative contrast value (-0.87). In Experiment 2, only the pixels in the inducing dots were set to the maximum displayable contrast value. For the remaining image pixels, the contrast energy was manipulated across trials using a 2-down, 1-up adaptive staircase procedure in order to maintain constant performance, as well as obtain contrast energy detection thresholds. Contrast energy is defined as the sum of the squared pixel contrast values multiplied by the area of an individual pixel, i.e.:

(1) $$E=\overset{n}{\underset{i=1}{\Sigma }}C_{i}^{2}a,$$

where *n* is the number of image pixels, *C* is the contrast at each pixel, and *a* is the area of an individual pixel, expressed in degrees squared (Tjan et al., 1995).

In addition, Gaussian white contrast noise of a fixed variance (σ^2^ = 0.16, NSD = 2.7e^-4^) was added to all pixels (except for the inducing dots) within a 200 pixel × 200 pixel (3.27° × 3.27°) region centered at the central dot. Noise samples that exceeded ±2 standard deviations were discarded and replaced with fresh samples. This insured that the noise distribution retained its normal shape while removing any values that exceeded the maximum displayable positive and negative contrast values. The stimulus duration was 43 frames (~500 ms).

### THRESHOLD ESTIMATION

Contrast energy detection thresholds in Experiment 2 were estimated by fitting Weibull psychometric functions to the staircase data in each condition and interpolating to find the contrast energy value that corresponded to 71% correct performance. Bootstrap simulations (Efron and Tibshirani, 1993) were carried out in order to estimate the error associated with each threshold estimate (500 simulated experiments per threshold).

### PROCEDURE

In Experiment 1, on each trial either the Small Inducers stimulus, Large Inducers stimulus, or a single isolated central dot stimulus with no surrounding inducers (No Inducers) was displayed in the center of the CRT (all were noise-free and set to the maximum displayable negative contrast). A second isolated dot figure (also set to the maximum displayable negative contrast) simultaneously appeared on the display and was offset 200 pixels (3.27°) to the right and 200 pixels down from the central stimulus. On half of the trials, the size of the offset dot was initially set at 15 pixels (0.25°); on the other half of the trials, the size of the offset dot was initially set at 68 pixels (1.11°; chosen randomly on each trial with equal probability). Once the stimuli were displayed, the observer was instructed to use two keys to manipulate the size of the offset dot so that it appeared to match the size of the central dot. Three observers completed 20 trials in each stimulus condition (i.e., Small Inducers, Large Inducers, and No Inducers). Trials were blocked by condition, with one observer completing each of the three conditions first.

In Experiment 2, either the Small Inducers or Large Inducers stimulus was displayed in the center of the CRT (a No Inducers condition was not included due to the complicating effects of spatial uncertainty at low contrast in the absence of inducers). The stimuli were shown in high contrast noise, and the contrast energy of the central dot was varied across trials to keep performance at roughly 71% correct throughout the experiment. On half of the trials, the central dot was actually present; on the remaining half of the trials, the dot was absent (randomly chosen). The observer’s task was to indicate whether or not the central dot had been present on a given trial. Accuracy feedback was given in the form of a high or low beep. Each observer from Experiment 1 participated in 10,000 trials in both stimulus conditions, measured over the course of approximately 3 weeks. Trials were blocked by condition, with two observers completing the Large Inducers condition first and the other observer completing the Small Inducers condition first.

## RESULTS

### EXPERIMENT 1: SUBJECTIVE RATINGS

**FIGURE 2:**
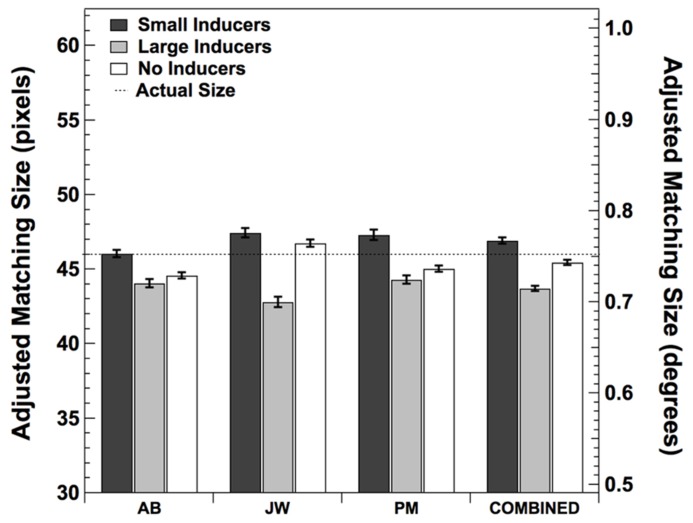
**Mean adjusted size matches in each condition from Experiment 1.** Error bars correspond to ±2 standard errors of the mean.

The purpose of Experiment 1 was to verify the presence and measure the magnitude of the subjective size-contrast illusion produced by the Ebbinghaus–Titchener patterns shown in **Figure 1**. Three observers repeatedly adjusted an isolated circle to match the perceived size of the central dot in each stimulus condition. The mean adjusted matching sizes for each observer as well as the mean values across observers are shown in **Figure 2**. These data show there was a consistent effect of the presence of the inducers, with Large Inducers producing smaller estimates than Small Inducers and No Inducers falling in between. A one-way repeated measures ANOVA revealed a significant effect of condition [*F*(2,2) = 7.74, *p* < 0.05]. *Post hoc* comparisons using the Tukey HSD test indicated that the mean estimate in the Small Inducers condition was significantly greater than the mean estimate in the Large Inducers condition (*p* < 0.05). There were no significant differences between the mean estimates in the No Inducers condition and either the Small or Large Inducers conditions. Thus, Experiment 1 established that our stimuli produced significant size-contrast illusions for all three of our observers.

### EXPERIMENT 2: BEHAVIORAL PERFORMANCE, EFFICIENCY AND CLASSIFICATION IMAGES

Experiment 2 was designed to explore what impact the Ebbinghaus–Titchener size-contrast illusion has on behavioral performance, the efficiency of information use and observers’ classification strategies when they are asked to perform a task that directly relies on the features that are perceptually distorted by the illusion. We asked the same three observers that participated in Experiment 1 to perform a detection task, in which the contrast of the central dot of the Ebbinghaus–Titchener figure was varied across trials in order to measure contrast detection thresholds in each condition. The stimuli were shown in high contrast Gaussian white noise (with the exception of the locations where the inducers appeared, which were always noise-free and shown at the maximum displayable negative contrast).

**FIGURE 3:**
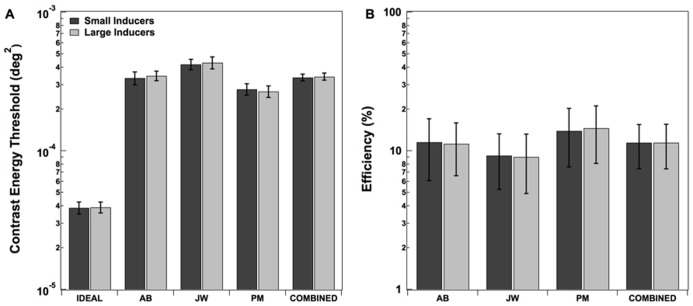
**Contrast energy thresholds (A) and efficiencies (B) in each condition of Experiment 2.** Error bars for individual observers correspond to ±2 standard deviations, estimated by bootstrap simulations. Error bars on combined thresholds correspond to ±2 standard errors of the mean.

Detection thresholds for all three human observers as well as the mean values across observers are shown in **Figure 3A**. The performance of a statistically optimal or “ideal observer” was also measured in each condition (Green and Swets, 1966; Braje et al., 1995). Such an observer uses a decision rule that maximizes the posterior probability of choosing whether or not the central dot was present (see Braje et al., 1995 for a detailed description the ideal decision rule in a detection task). The ideal observer’s thresholds were estimated by carrying out Monte Carlo simulations in each condition for the same number of trials as the human observers (10,000). The ideal observer’s thresholds are plotted in the leftmost side of the **Figure 3A**. Finally, the ratio of ideal to human threshold (*efficiency*) was computed for each human observer in each condition (**Figure 3B**). As expected, the ideal observer’s thresholds were the same for the Large and Small Inducers conditions. Although human thresholds differed by about an order of magnitude from those of the ideal observer (yielding efficiencies of ~10%), there was no discernable effect of inducer condition on human efficiency. A two-tailed paired-samples *t*-test confirmed that the effect of condition for the human observers was not statistically significant; *t*(2) = -0.68, *p* = 0.57.

In addition to the thresholds and efficiencies, we used the noise presented over the course of the experiment to generate classification images for each observer in each condition (Ahumada and Lovell, 1971; Ahumada, 2002; Murray et al., 2002). Classification images were computed by first sorting the noise for a given observer in a given condition according to the Stimulus (*present*, *absent*)–Response (*present*, *absent*) combination. Next, the noise was averaged within each stimulus–response (S–R) pairing and then combined to form a single *classification image* *C*:

(2) $$C=\left(S_{absent}R_{present}+S_{present}R_{present}\right)-\left(S_{absent}R_{absent}+S_{present}R_{absent}\right)$$

**FIGURE 4:**
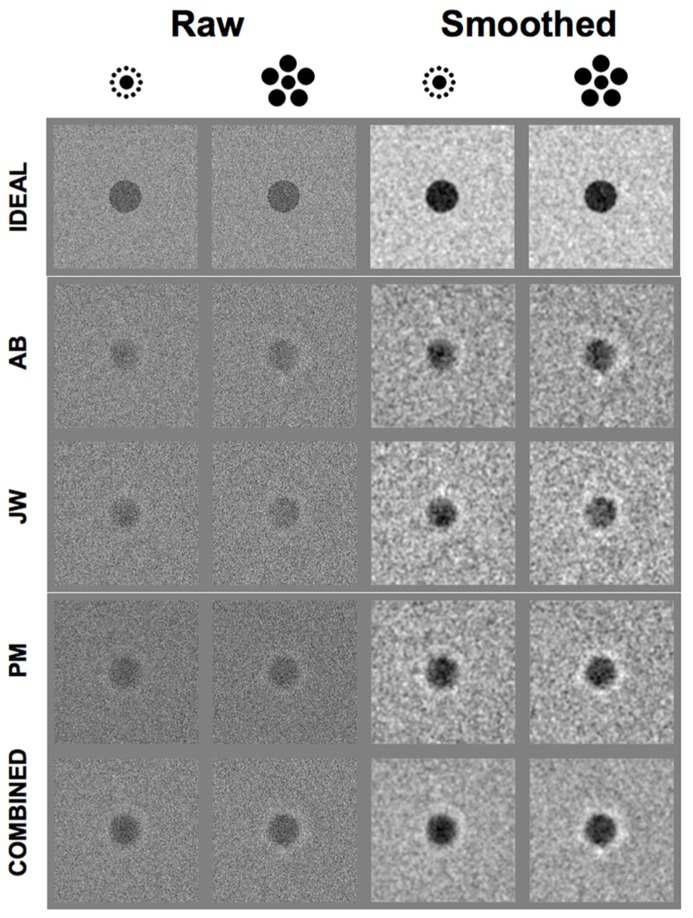
**Raw (left two columns) and smoothed (right two columns) classification images for the ideal observer, three human observers and the combined data across all three human observers in each condition of Experiment 2 (see text for details)**.

The resulting classification images show the relative weight assigned to each pixel in the display by the observer over the course of the experiment. The classification images in each condition for each human observer as well as the ideal observer are shown in the left two columns of **Figure 4**. The bottom row of these columns also shows the classification images generated by combining all of the trials across the three human subjects in each condition. The right two columns of **Figure 4** show the same classification images smoothed by a small (7 pixel × 7 pixel, 0.11° × 0.11°) convolution kernel. Note that the regions where the inducing elements appeared are not noise-free. These regions were simply populated by random noise samples when computing the classification images. This was done in order to avoid inducing the illusion itself when visualizing the data. That is, presenting the classification images with the inducing element regions set to some constant value (e.g., 0), would potentially affect the perceived size of the central regions, and thus make it difficult to visually compare them across conditions. Adding random noise samples to these regions when generating the classification images allows them to blend naturally into their neighboring background regions.

These data show that the human observers adopted a very specific strategy in both conditions. Namely, each human observer evaluated the contrast of both the inner region (where the central dot appeared) as well as a circular region that surrounded the central dot. In addition, observers responded differentially to contrast in these two regions. Specifically, if the contrast of the noise was negative in the region of the central dot, observers were more likely to respond “present” (or, if the contrast of the noise in this region was positive, observers were more likely to respond “absent”). However, the opposite was true in the annular region that surrounded the central dot: if the contrast of the noise was positive in this region, observers were more likely to respond “present” (or, if the contrast of the noise in this region was negative, observers were more likely to respond “absent”). Note that this strategy of using an annular region surrounding the central dot is not ideal: the ideal observer uses only the central dot region where the stimulus was actually present; the surrounding region carries no physical information for performing the task. Similar center-surround effects have been reported for tasks requiring observers to detect or discriminate a centralized target in noise (e.g., Shimozaki et al., 2005).

The results of the classification image analysis are consistent with the idea that, unlike the ideal observer, human observers were comparing the contrast within the region of the central dot to the contrast immediately surrounding the central dot region in order to make their classification decisions. However, this center-surround effect appears to be independent of the presence of the Large and Small Inducers. To explore the effect of inducer size more closely, we took advantage of the circular-symmetric shape of the central portion of our stimuli and radially averaged the raw classification images (Abbey and Eckstein, 2002, 2007). This produced a set of one-dimensional classification images that revealed the weights observers assigned to each distance from the midpoint of the central dot in each condition.

**FIGURE 5:**
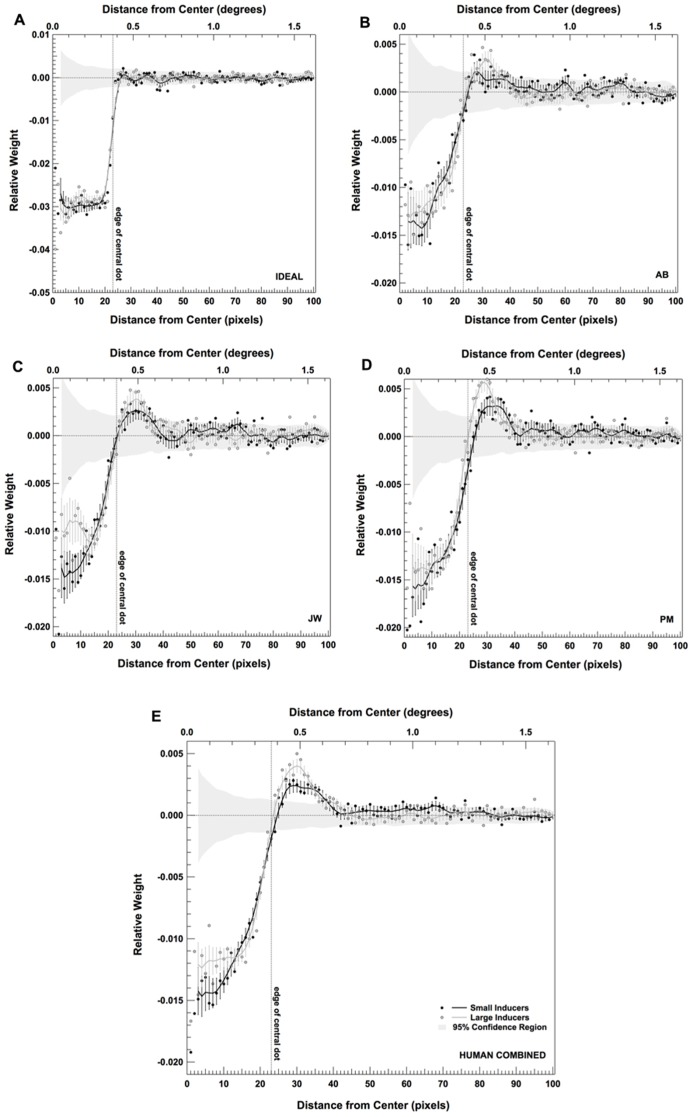
**Radially averaged classification images for the ideal observer (A), three human observers **(B–D)** and the combined data across all three human observers **(E)** in each condition of Experiment 2.** Error bars correspond to ±2 standard deviations, estimated by bootstrap simulations. The gray band shows a region ±2 standard deviations around what would be expected from a purely random classification image for the same number of trials (estimated by bootstrap simulations; see text for details).

The results of this radial classification image analysis are shown in **Figure 5**. **Figures 5A–D** plots the results for an individual observer in each condition (including the ideal observer; **Figure 5A**). **Figure 5E** plots the results when the data are combined across all three human observers. Individual points in each plot correspond to the raw classification image weights. The solid lines correspond to the average classification image generated by running 500 bootstrap simulations (generated by sampling the data in each condition with replacement for each observer) and then smoothing these images with a convolution kernel. The error bars on each smoothed curve correspond to ±2 standard deviations, calculated from the bootstrap simulations. Finally, the dashed vertical line in each plot shows the location of the edge of the central dot. These data reveal that, although the spatial extent of the regions used by human observers was similar across conditions, the relative weights assigned to the central and the surrounding regions were markedly different. Specifically, all three human observers tended to place relatively more weight upon the central dot region in the presence of Small Inducers and relatively more weight on the surrounding region in the presence of Large Inducers.

We ran two sets of statistical analyses in order to verify these effects. The first was a parametric test for the overall statistical significance of (a) the difference between each raw radial classification image and the null hypothesis of zero correlation; and (b) the difference between the raw radial classification images obtained in the presence of Small vs. Large Inducers for each observer and the data combined across observers. We used the single-sample Hotelling *T*^2^ statistic to test against the null hypothesis of zero correlation and the independent two-sample Hotelling *T*^2^ statistic to test for significant differences between inducer conditions (for details on computing Hotelling *T*^2^ statistics, see Abbey and Eckstein, 2002; Eckstein et al., 2002; Shimozaki et al., 2005). The results of these tests are shown in **Table 1** (single-sample tests) and **Table 2** (two-sample tests). These data confirm that the overall classification images for all observers in both conditions significantly differed from a zero-correlation classification image, and that the overall difference between the Small and Large Inducer classification images was highly significant for all observers.

**Table 1:** Degrees of freedom, *F* values and *p* values obtained from the single-sample Hotelling *T*^2^ statistic, testing the radial classification images obtained for each human observer and the combined data across observers in each condition against the null hypothesis of zero correlation.

Observer	df numerator	df denominator	*F* value	*p* Value
**Small Inducers vs. zero**
AD	99	9901	149.48	<0.0001
JW	99	9901	159.84	<0.0001
PM	99	9901	207.19	<0.0001
COMBINED	99	29901	459.71	<0.0001
**Large Inducers vs. zero**
AD	99	9901	158.28	<0.0001
JW	99	9901	137.10	<0.0001
PM	99	9901	201.94	<0.0001
COMBINED	99	29901	455.02	<0.0001

**Table 2:** Degrees of freedom, *F* values and *p* values obtained from the independent two-sample Hotelling *T*^2^ statistic, testing for the difference between the radial classification images obtained for each human observer and the combined data across observers with Large and Small Inducers.

Large Inducers vs. Small Inducers	
Observer	df numerator	df denominator	*F* value	*p* Value
AD	99	19900	23.54	<0.0001
JW	99	19900	27.13	<0.0001
PM	99	19900	29.72	<0.0001
COMBINED	99	59900	33.21	<0.0001

We next gauged the likelihood that the weights at each location deviated significantly from what would be expected purely by chance by generating a series of classification images that were created by randomly choosing noise images on each trial of the experiment. Specifically, these classification images were created by replacing the noise samples generated in our experiment with newly generated noise samples and re-computing the classification images. We generated 200 of these random classification images for the individual subject data sets (10,000 trials) and another 200 for the collapsed data set (30,000 trials). We then computed the mean and standard deviation across these replications in order to generate the gray band shown in each panel of **Figure 5**. Thus, this band represents ±2 standard deviations around the mean randomly generated classification image. These simulations show that the human classification image weights within and directly surrounding the central dot fell well outside of this region (with the exception of the locations corresponding to the border between the two regions).

**FIGURE 6:**
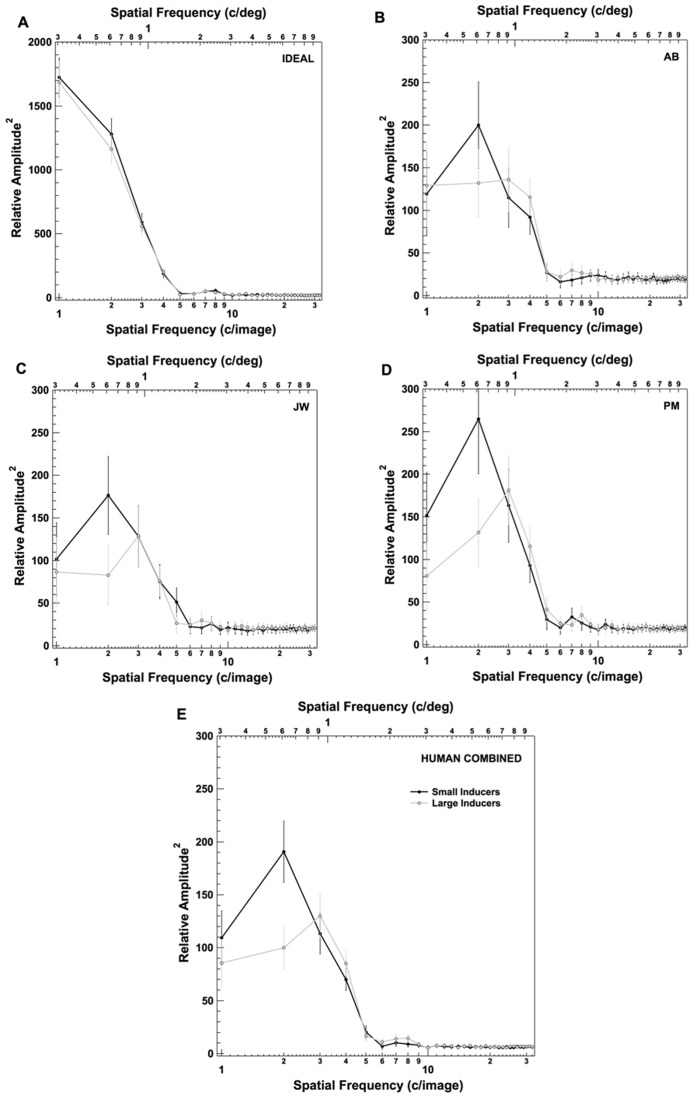
**Frequency domain representation of the raw classification images for the ideal observer (A), three human observers **(B–D)** and the combined data across all three human observers **(E)** in each condition of Experiment 2.** Error bars correspond to ±2 standard deviations (estimated by bootstrap simulations; see text for details).

In addition to this spatial classification image analysis, we also explored the effects of inducer size on observer’s use of information across spatial frequencies. Specifically, we transformed each of the classification images shown in the left two columns of **Figure 4** into the spatial frequency domain, and computed the average squared amplitude at each spatial frequency in each image (**Figure 6**). As in **Figure 5**, **Figures 6A–D** plots the results for an individual observer in each condition (including the ideal observer; **Figure 6A**). **Figure 6E** plots the results when the data are combined across all three human observers. Individual points in each plot correspond to the average squared amplitude in the classification image at a particular spatial frequency. The error bars on each point correspond to ±2 standard deviations, computed by running 500 bootstrap simulations (generated by sampling the data in each condition with replacement for each observer). These data reveal that observers adopted a strategy that involved placing relatively more weight on slightly higher frequencies in the presence of Large Inducers (peak at ~~6 c/deg in the presence of Small Inducers and ~9 c/deg in the presence of Large Inducers).

## DISCUSSION

The goal of our experiments was to explore the information processing correlates of the Ebbinghaus–Titchener size-contrast illusion. In Experiment 1, we replicated the results of many previous experiments by demonstrating the subjective reality of this illusion. In Experiment 2, we asked observers perform a detection task with the same stimuli used in Experiment 1, albeit embedded in high contrast visual noise. By comparing observers’ contrast detection thresholds in this task to that of an ideal observer, we found that the efficiency with which observers used information did not depend upon the size of the inducing elements. By computing the correlation between the noise contrast at each pixel and the observers’ responses across trials, we found that observers tended to place relatively more weight upon the region surrounding the inner dot in the presence of Large Inducers and relatively more weight upon the region inside the inner dot in the presence of Small Inducers. We also found that observers tended to place relatively more weight upon slightly higher frequencies in the presence of Large Inducers (i.e., ~9 c/deg) and relatively more weight upon slightly lower frequencies in the presence of Small Inducers (i.e., ~6 c/deg).

So how do we interpret these findings? First, consider the finding that efficiency was unaffected by the size of the inducing elements. On the one hand, the subjective ratings given by observers in Experiment 1 showed that observers’ judgments of size are farther from veridical in the presence of Large than Small Inducers. In addition, the tendency of human observers to assign relatively greater weight to the center and relatively less weight to the surround in the presence of Small Inducers is more similar to the weights used by the ideal observer, which would predict efficiency should be greater in the presence of Small than Large Inducers (Murray et al., 2005). However, there are several reasons why we might not expect to see such variations in efficiency across conditions in Experiment 2. First, there is no necessary relationship between an observer’s subjective experience of an illusion and their ability to perform a task with the stimuli that produce the illusion. That is, it is unclear how the misjudgments in perceived size found in Experiment 1 should map on to an observer’s ability to make use of information in Experiment 2. The most we can ultimately hope for is that there may be some correlation between the two (Teller, 1984). Second, the task we asked observers perform does not directly rely upon the precision of size judgments, only the ability to detect the presence of the central dot. As such, it is unclear that greater misjudgments in size would negatively affect performance in such a task. And finally, the prediction that greater similarity between the human and ideal classification images should lead to greater efficiency assumes a number of other factors know to effect efficiency are invariant across conditions (e.g., internal noise, point-wise non-linearities; Murray et al., 2005). More detailed measurements and analyses than those reported here would be required in order to properly test this prediction.

**FIGURE 7:**
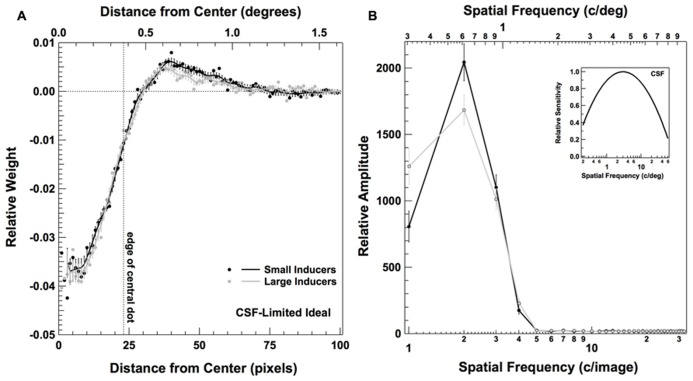
**Classification images for a CSF-limited ideal observer in Experiment 2.** Panel **(A)** plots the radially averaged classification image, as described in **Figure 5**; panel **(B)** plots the frequency domain representation of the raw classification image, as described in **Figure 6**. Inset figure in **(B)** plots the CSF used to limit the performance of the ideal observer (see text for details).

Despite the equivocal nature of the efficiencies obtained in Experiment 2, observers nevertheless exhibited the use of a markedly different strategy in the presence of Large and Small Inducers. So why observers might have adopted such different strategies within different contexts? One potential source of this effect could be the spatial frequency filtering that takes place during the early stages of visual processing (Geisler, 1989). We explored this possibility by building an ideal observer that was ideal in all respects, with the exception that it was limited by the foveal contrast sensitivity function (CSF) of a normal adult human (inset of **Figure 7B**). The CSF was generated from the fits reported in Watson (2000). The CSF-limited ideal observer analysis was carried out in a fashion similar to that described by Chung et al. (2002) and Nandy and Tjan (2008). Specifically, the CSF was applied to both the noise-free signals (with the inducing elements present) as well as the noise-free templates (without the inducing elements present) in the frequency domain in each condition. On each trial, unfiltered white noise of the same variance as used in the original experiments was added to the filtered signal, and the filtered templates were used to compute the likelihoods for each alternative (i.e., present, absent). All other aspects of the CSF-limited ideal observer analysis were the same as those used for the original ideal observer analysis.

**Figure 7** shows the classification images obtained from a simulated experiment carried out with our CSF-limited ideal observer performing the same detection task and for the same number of trials as our human observers. **Figure 7A** plots the radially averaged classification image, computed in the same fashion as the plots in **Figure 5**; **Figure 7B** shows the Fourier representation of the classification image, computed in the same fashion as the plots in **Figure 6**. Interestingly, these data reveal that the center-surround weighting in the human classification images is well predicted by the filtering characteristics of the human visual system. That is, unlike the true ideal observer, our human observers and the CSF-limited ideal observer both give weight to the area directly surrounding the central dot as well as the area within the central dot. Despite these similarities, there appear to be no discernable differences in the weighting of the center relative to the surround in the presence of Large vs. Small Inducers for the CSF-limited ideal observer. We also do not see the characteristic shift toward weighting slightly higher spatial frequencies in the presence of Large relative to Small Inducers that we found with our human observers. Thus, although the human CSF accurately predicts the gross center-surround characteristics of the human observers’ classification images, the results of our simulation suggest it is unlikely that the human observers’ tendency to differentially weight the center and surround in the presence of different sized inducers was due to the spatial frequency filtering that takes place during the early stages of visual processing. The connection between the variations in perceived size of the central element and the differential weighting of the center and surround thus remains unclear.

Of course, it is always possible that the magnitude of the Ebbinghaus–Titchener size-contrast illusion is greatly reduced or even non-existent when the central dot is presented at low contrast in large amounts of pixel noise, as it was in our experiments. One argument against this idea is that fact that our response classification analyses showed that there were significant differences in how observers made use of information within the context of large and small inducers – an effect that is presumably related to the subjective experience of the illusion. However, the results of at least one study suggest that there may in fact be some effect of the relative contrast of the central and surrounding dots in the magnitude of the illusion. Jaeger and Pollack (1977) asked participants to make subjective judgments of the size of the central dot when (a) the inducing dots and the central dot were both “black” and (b) the inducing dots were “black” and the central dot was “gray” (i.e., relatively lower in contrast). Stimuli were shown against a uniform “white” background, and the inducing elements were either larger or smaller than the central dot (the actual luminance or contrast values used in the experiment were not specified). They found that the magnitude of the illusion was reduced when the central dot was gray relative to when it was black when the inducing dots were large; however, they found the opposite effect when the inducing elements were small: the magnitude of the illusion increased when the central dot was gray relative to when it was black.

Although the above study suggests that there may be some relationship between the relative contrasts of the central and surrounding elements and the magnitude of the Ebbinghaus–Titchener illusion, the asymmetric effects of inducer size and the lack of specification of the luminance and contrast levels make the result somewhat difficult interpret. As such, we decided to address this issue experimentally by having a new set of six observers make subjective size ratings with low contrast stimuli in the presence of high contrast noise, modeled closely after the conditions experienced by our observers when participating in Experiment 2. Specifically, we averaged the contrast energy thresholds obtained for our original three observers and doubled this value, in order to place it just over detection threshold. We then used this value to set the contrast of the inner dot of the illusion figure, in each of the conditions described in the Experiment 1 (i.e., Large Inducers, Small Inducers, and No Inducers). We also added high contrast Gaussian noise to the figure, in the same manner and at with the same variance as described in Experiment 2. A new sample of noise was added to the figure for every trial of the experiment (15 trials in each condition), and the offset comparison dot that observers were asked to adjust remained high in contrast and noise-free. Each observer was tested in these three conditions, as well as the same three high-contrast, no-noise conditions originally tested in Experiment 1 (six conditions in all). The order of the conditions was randomized for each observer. All other aspects of the experiment were the same as described in Experiment 1.

**FIGURE 8:**
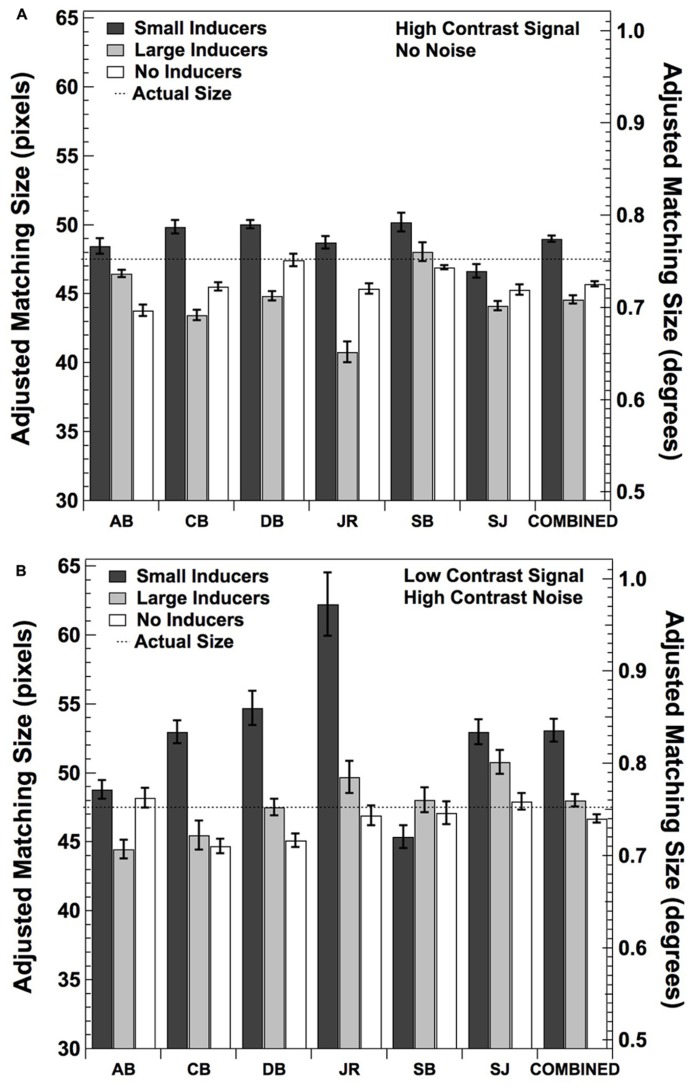
**Mean adjusted size matches for six new observers and the data combined across all observers under the same conditions as in Experiment 1 (A) and Experiment 2 (B).** Error bars correspond to ±2 standard errors of the mean.

The results of this subjective rating experiment are shown in **Figure 8**. **Figure 8A** shows the results for the conditions that are the same as Experiment 1 (i.e., high contrast stimuli with no added noise). All observers exhibited the characteristic effect of judging the central dot to be relatively greater in size in the context of small than large inducers, and four of the six observers judged the size of the central dot to fall somewhere in between in the absence of inducers. A one-way repeated measures ANOVA revealed a significant effect of condition [*F*(2,5) = 12.53, *p* < 0.01]. *Post hoc* comparisons using the Tukey HSD test indicated that the mean estimates were significantly greater in the Small Inducers condition than the Large Inducers condition (*p* < 0.01) as well as the No Inducers condition (*p* < 0.05), with no significant difference between the Large Inducers and No Inducers conditions.

**Figure 8B** shows the results when the middle dot was low in contrast and embedded in high contrast noise. All but one observer (SB) exhibited the characteristic effect of judging the central dot to be relatively greater in size in the context of small than large inducers. Surprisingly, only one observer (AB) judged the size of the central dot to fall somewhere in between these sizes in the absence of inducers; the remaining five observers judged the size of the central dot to be *smallest* in the absence of inducers. This result is consistent with the asymmetric effects of brightness reported by Jaeger and Pollack (1977). A one-way repeated measures ANOVA again revealed a significant effect of condition [*F*(2,5) = 5.51, *p* < 0.05]. *Post hoc* comparisons using the Tukey HSD test indicated that the mean estimates were significantly greater in the Small Inducers condition than the Large Inducers and No Inducers conditions (*p* < 0.05), with no significant difference between the Larger Inducers and No Inducers conditions. Finally, A 2 (stimulus contrast condition) × 3 (inducer condition) two-factor ANOVA with repeated measures on both factors showed that there was a significant effect of inducer condition [*F*(5,2) = 11.11, *p* < 0.01] with no significant effect of stimulus contrast condition [*F*(5,1) = 3.53, *p* = 0.11] nor a significant inducer condition × stimulus contrast condition interaction [*F*(5,2) = 1.09, *p* = 0.37]. Taken together, these results demonstrate that the Ebbinghaus–Titchener size-contrast illusion is relatively unaffected by the presentation of the central dot at a low level of contrast within high contrast pixel noise, and strongly suggest that our original three observers were experiencing the size illusion under the conditions used in Experiment 2.

## CONCLUSION

The results of our experiments offer some interesting new insights into the information processing correlates of the Ebbinghaus–Titchener size-contrast illusion. Namely, the subjective size of the central element in the illusion appears to be related to the amount of weight observers assign to the areas within and directly surrounding the central element as well as the range of spatial frequencies that they rely upon when they are asked to perform a simple detection task. We were unable to account for this effect by a simple model that incorporates the overall spatial frequency filtering characteristics of early visual processing, as summarized by the foveal CSF of a normal human adult. Given these results, it may be tempting to conclude that the effects we have observed are due to the operation of processes involved with making higher-level judgments about the relative sizes of objects (e.g., Massaro and Anderson, 1971; Coren and Girgus, 1978; Coren and Enns, 1993). However, it is still possible that a more detailed front-end model (e.g., Chirimuuta et al., 2003) that incorporates additional aspects of the early stages of visual processing, such as oriented V1 receptive fields, parafoveal variations in contrast sensitivity, and cortical magnification, might make predictions not captured by simply incorporating the overall CSF, and these predictions may map more directly on to the results of our classification image analyses.

**FIGURE 9:**
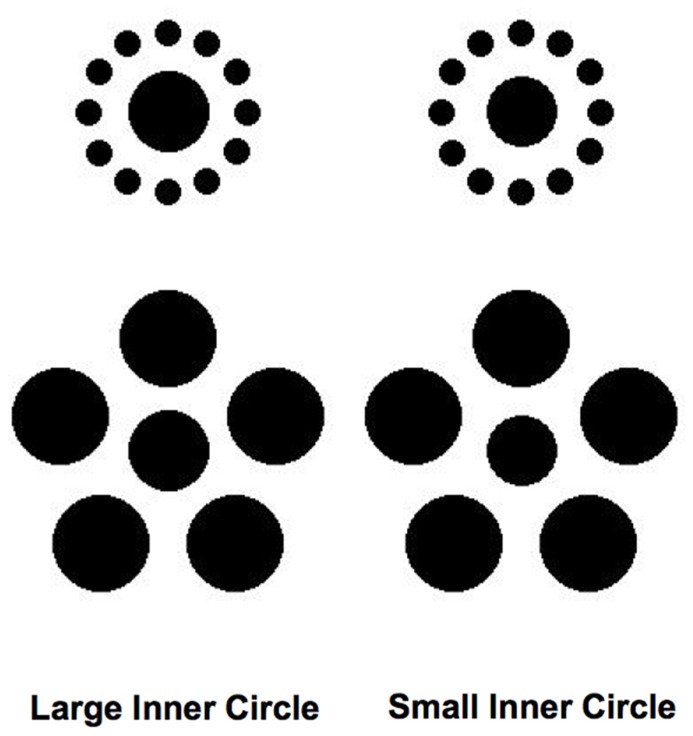
**Hypothetical stimuli that could be used in an experiment that would involve having observers discriminate between the sizes of the inner circles rather than detecting their presence (see text for details)**.

Finally, although we chose to use a detection task in our experiments for its relative simplicity, an interesting future direction would be to carry out similar experiments using tasks that might rely more directly upon an observer’s ability to make judgments about relative size. **Figure 9** illustrates a task and set of stimuli one might use in such a hypothetical experiment. In this case, an observer would be asked to determine which of two central dots that slightly differ in size had appeared on a given trial, in the presence of either large or small inducing elements. It is possible that such a task would tap more directly into the same underlying processes that lead to the misperception of size associated with the subjective experience of the Ebbinghaus–Titchener illusion. We are currently exploring these and other possibilities.

## Conflict of Interest Statement

The author declares that the research was conducted in the absence of any commercial or financial relationships that could be construed as a potential conflict of interest.
